# Spatio-temporal variation of malaria hotspots in Central Senegal, 2008–2012

**DOI:** 10.1186/s12879-020-05145-w

**Published:** 2020-06-17

**Authors:** Sokhna Dieng, El Hadj Ba, Badara Cissé, Kankoe Sallah, Abdoulaye Guindo, Boukary Ouedraogo, Martine Piarroux, Stanislas Rebaudet, Renaud Piarroux, Jordi Landier, Cheikh Sokhna, Jean Gaudart

**Affiliations:** 1 Aix Marseille Univ, IRD, INSERM, SESSTIM, Marseille, France; 2 Ecole des Hautes Etudes en Santé Publique, Rennes, France; 3 UMR VITROME, Campus International IRD-UCAD de l’IRD, Dakar, Sénégal; 4 Institut de Recherche en Santé, de Surveillance Épidémiologique et de Formation (IRESSEF) Diamniadio, Dakar, Sénégal; 5 AP-HP, Hôpital Bichat, Unité de Recherche Clinique PNVS, Paris, France; 6 Research and Training Center - Ogobara K Doumbo, FMOS-FAPH, Mali-NIAID-ICER, Université des Sciences, des Techniques et des Technologies de Bamako, Bamako, Mali; 7 Direction des Systèmes d’Information en santé, Ministère de la santé, Ouagadougou, Burkina Faso; 8 French Armed Forces Center for Epidemiology and Public Health (CESPA), Marseille, France; 9 APHM, Assistance Publique - Hôpitaux de Marseille, Marseille, France; 10 Hôpital Européen, Marseille, France; 11 Sorbonne Université, INSERM, Institut Pierre-Louis d’Epidémiologie et de Santé Publique, AP-HP, Hôpital Pitié-Salpêtrière, Paris, France; 12 Aix Marseille Univ, APHM, INSERM, IRD, SESSTIM, Hop Timone, BioSTIC, Biostatistic & ICT, Marseille, France

**Keywords:** Spatial clusters, Spatio-temporal dynamic, Malaria hotspots, Non-linear associations, Geostatistical analyses

## Abstract

**Background:**

In malaria endemic areas, identifying spatio-temporal hotspots is becoming an important element of innovative control strategies targeting transmission bottlenecks. The aim of this work was to describe the spatio-temporal variation of malaria hotspots in central Senegal and to identify the meteorological, environmental, and preventive factors that influence this variation.

**Methods:**

This study analysed the weekly incidence of malaria cases recorded from 2008 to 2012 in 575 villages of central Senegal (total population approximately 500,000) as part of a trial of seasonal malaria chemoprevention (SMC). Data on weekly rainfall and annual vegetation types were obtained for each village through remote sensing. The time series of weekly malaria incidence for the entire study area was divided into periods of high and low transmission using change-point analysis. Malaria hotspots were detected during each transmission period with the SaTScan method. The effects of rainfall, vegetation type, and SMC intervention on the spatio-temporal variation of malaria hotspots were assessed using a General Additive Mixed Model.

**Results:**

The malaria incidence for the entire area varied between 0 and 115.34 cases/100,000 person weeks during the study period. During high transmission periods, the cumulative malaria incidence rate varied between 7.53 and 38.1 cases/100,000 person-weeks, and the number of hotspot villages varied between 62 and 147. During low transmission periods, the cumulative malaria incidence rate varied between 0.83 and 2.73 cases/100,000 person-weeks, and the number of hotspot villages varied between 10 and 43. Villages with SMC were less likely to be hotspots (OR = 0.48, IC95%: 0.33–0.68). The association between rainfall and hotspot status was non-linear and depended on both vegetation type and amount of rainfall. The association between village location in the study area and hotspot status was also shown.

**Conclusion:**

In our study, malaria hotspots varied over space and time according to a combination of meteorological, environmental, and preventive factors. By taking into consideration the environmental and meteorological characteristics common to all hotspots, monitoring of these factors could lead targeted public health interventions at the local level. Moreover, spatial hotspots and foci of malaria persisting during LTPs need to be further addressed.

**Trial registration:**

The data used in this work were obtained from a clinical trial registered on July 10, 2008 at www.clinicaltrials.gov under NCT00712374.

## Background

Of the 435,000 deaths attributed to malaria worldwide in 2017, 93% were recorded in sub-Saharan Africa [1]. In Senegal, annual malaria incidence rose from 14 to 25.94 cases/1000 person-years between 2009 and 2017, despite the strengthening of control strategies. In 2017, malaria incidence varied between 0.4 (Saint-Louis) and 473.9 cases/1000 person-years (Kedougou) across the country’s 72 health districts [2].

It is now known that spatial heterogeneity of malaria distribution reduces the effectiveness of malaria control. Thus, this spatial heterogeneity may contribute to the persistence of transmission at a significant level [3]. The detection of heterogeneity patterns in malaria endemic areas is therefore becoming an important element in recent approaches that seek to identify transmission bottlenecks [4–13]. Variations in heterogeneity have been observed even at a very local scale—for example, within 8-km radius areas in Kenya [7], at the village level in Senegal (KeurSocé) [14], and at the household level in Tanzania (Korogwe District) and Mali (Bandiagara) [9, 15]. Central Senegal has a surveillance system which provides data on malaria cases and population at the village level [16]. Moreover, the heterogeneous distribution of malaria has been shown to depend on a series of environmental factors that favour vectors development and host-vector interactions [17, 18].

In view of these findings, the WHO (World Health Organization) recommends developing targeted strategies aimed at accelerating the malaria elimination process [5, 19]. The first step to ensure the effectiveness of these strategies is to accurately identify geographical areas of greatest risk, the so-called hotspots, where they are expected to exert a stronger impact on malaria transmission. Indeed, hotspots can maintain transmission during the dry season, and they can be the source of epidemic episodes during the rainy season [5].

However, while some locations exhibit constant hotspot status, certain hotspots can be unstable over time [8]. This instability adds a layer of complexity to the process of malaria transmission, and may consequently hinder the effectiveness of prevention strategies. Yet, while a number of studies on malaria have explored the environmental factors that may influence spatial hotspot distribution [5, 7, 8, 20, 21], few have attempted to explain the spatio-temporal dynamics of malaria hotspots [22].

The aim of this work was to describe the spatio-temporal variation of malaria incidence hotspots in villages of central Senegal and to identify the meteorological, environmental, and preventive factors that influence this variation.

## Methods

### Study area and dataset

The study area included 575 villages that spanned 2 health districts, Bambey and Fatick, which are located in west-central Senegal [16]. The total population of the study area was approximately 500,000. The median population of the villages was 499 [interquartile range 233; 1029]. Each of the village was linked to one of the 38 health posts covering the 2 health districts. The median number of villages linked to each health post was 11 [interquartile range 8; 19].

In 2017, 1.2 cases/1000 person-years were recorded in the Bambey health district and 2.1 cases/1000 person-years were recorded in the Fatick health district [2].

The trial of seasonal malaria chemoprevention (SMC) was implemented in this area from 2008 to 2010 [16, 23–25]. The SMC protocol consisted in administering a combination of sulfadoxine-pyrimethamine and amodiaquine to children (under 5 years of age in 2008 and under 10 years of age in 2009–2010) once a month from September to November. As part of this trial [16, 25], a surveillance system was put in place in each health post of the study area. A list of GPS-positioned villages was established for each health post. A census of the population in each village was conducted annually from January 2008 to December 2012. Malaria cases (detected by rapid diagnostic test) were reported daily with the village name and collected at the health post level. Malaria cases were aggregated per week in this study.

Vegetation data for the same period were derived from MODIS (Moderate-Resolution Imaging Spectroradiometer) rasters [26]. Four MODIS-derived vegetation types were present in the area: open shrublands, grasslands, croplands, and mixed vegetation. The latter consisted of a mosaic of croplands, forests, shrublands, and grasslands, in which no single component represented more than 60% of the landscape. For each village centre, a buffer zone of 0.55 km (550 m) radius was defined. This was the smallest radius at which we could operate the sensor data. The percentage of surface areas occupied by different types of vegetation within a 0.55 km radius buffer zone was calculated annually (Additional file 1), and the vegetation type covering the largest surface area was retained as the dominant type.

Total weekly rainfall was calculated for each village using daily rainfall amounts (mm) that were derived from the Tropical Rainfall Measuring Mission (TRMM) and extracted from the NASA Goddard Earth Sciences website with a 0.25 degree resolution [27].

### Statistical methods

First, we conducted a change-point analysis of the time series of weekly malaria incidence over the entire study area in order to detect High Transmission Periods (HTPs) and Low Transmission Periods (LTPs). As per this method, we identified the dates (referred to as change-points) associated with significant changes in the mean and variance of the malaria incidence rate. We chose to use the PELT (Pruned Exact Linear Time) algorithm and the MBIC (Modified Bayes Information Criterion) penalty criterion for convergence and optimization reasons [28–30].

Second, for each identified period, we searched for high-risk clusters (hotspots) using the SaTScan method developed by Kulldorff [31]. Following this approach, neighbouring villages were aggregated into groups with similar incidence using an elliptical window with variable size, centre, and rotation. Kulldorff’s statistics based on the likelihood ratio (Poisson model with a purely spatial analysis) were tested using a Monte Carlo algorithm (999 replicates). A hotspot was then selected when the incidence inside the window was significantly higher (*p* < 0.05) than the incidence outside. For a given transmission period, a village was defined as a hotspot if it belonged to a significant cluster detected by SaTScan.

Third, we used a generalized additive mixed model (GAMM) [32] to assess the spatio-temporal variation of hotspot status for each village according to successive transmission periods. This allowed us to assess the non-linear relation between variables and the hotspot risk, and to take into account the spatial effect.

Thus, a spline smoothing function of time by vegetation type, *f*_1_(*Time*, *by* = *Vegetation*) (eq.1), was used to estimate the temporal variation of hotspot risk according to vegetation type. Because the impact of rainfall on malaria can be modified by vegetation, a spline smoothing function of rainfall by vegetation type, *f*_2_(*Rain*, *by* = *Vegetation*) (eq.1), was used to estimate the variation of the rainfall effect on hotspot risk according to vegetation type.

We also included in the model a village-level binary variable, SMC, to estimate the effect of SMC interventions [16] on hotspot status.

A bivariate spline function of the geographical coordinates of villages [32], *f*_3_(*Longitude*, *Latitude*) (eq.1), was used to estimate the spatial variation of hotspot status, and thereby to obtain spatial interpolations for the entire study area.

The link between each village and its corresponding health post was expressed as a random effect of the “HealthPost” variable.

A first-order autoregressive correlation was integrated into the variance-covariance matrix to account for temporal autocorrelation. The final model was selected by minimizing the Akaike criterion.

1 $$ logit\ (P)=\ln \left(\frac{P}{1-P}\right)={\beta}_0+\beta \ast SMC+{f}_1\left( Time, by= Vegetation\right)+{f}_2\left( Rain, by= Vegetation\right)+{f}_3\left( Longitude, Latitude\right)+u\ast HealthPost+\varepsilon $$

where P is the probability (or risk) of a village being a hotspot, *β*_0_ was the intercept, *β* the associated fixed parameter estimating the SMC effect, *f*_1*,*_*f*_2_, and *f*_3_ the spline functions, *u* the random parameter associated with the *HealthPost*, and *ε* the residuals whose covariance matrix had a first-order autoregressive structure.

The results of the spline smoothing functions were plotted on the scale of logit(P), see eq. 1, where P is the probability (or risk) of a village being a hotspot. A factor X (i.e., the function of Time, Rainfall, or Location) has a changing effect according to the values taken (k): this is a non-linear effect. If the smoothed value, f(k), is positive, the risk of a village being a hotspot is increased at this value; if negative, the risk is decreased.

Statistical analyses were done with R 3.4.2 (The R Foundation for Statistical Computing, Vienna, Austria) (packages {changepoint} {mgcv}). Hotspot detection was performed with SaTScan 9.4 (Information Management Services Inc., Silver Spring, Maryland, USA). Hotspots maps were produced using QGIS 2.14.2 (Open Source Geospatial Foundation, Boston, USA) with statistical analysis results, Senegal shapefile [33] and landcover rasters [34] extracted from MODIS NASA.

## Results

### Temporal evolution of the malaria incidence

**Fig. 1:**
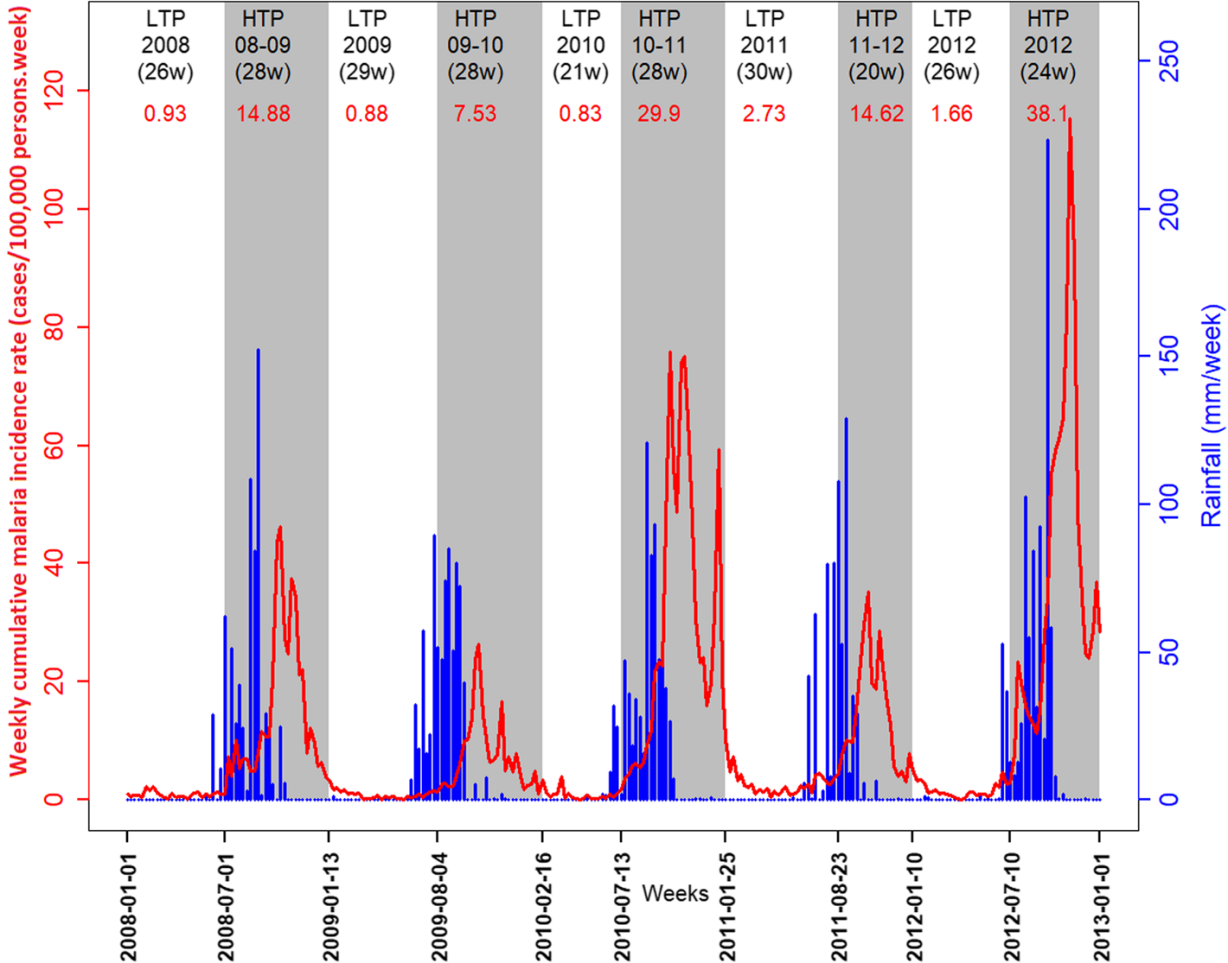
Evolution of weekly malaria incidence and rainfall through transmission periods. Malaria incidence (continuous red curve); High Transmission Periods (HTP, in grey) and Low Transmission Periods (LTP, in white) with their duration (weeks, in black) and cumulative incidence rates (red numbers); total weekly rainfall (in blue)

During 2008–2012, the malaria incidence rate for the entire area showed an annual resurgence dependent on rainfall (Fig. 1). The incidence rate peaks of epidemic periods ranged from 26.4 cases/100,000 person-weeks in 2009 (October) to 115.34 cases/100,000 person-weeks in 2012 (October). Low to very low incidences of malaria were recorded even during the driest and hottest seasons.

### Identification of malaria transmission periods

The change-point analysis helped to detect 5 LTPs and 5 HTPs (Fig. 1).

The HTPs (except for the last one) overlapped 2 consecutive years. Annual epidemics began in July or August and ended in January or February of the following year. The median HTP duration was 28 weeks. The 2012 HTP had the highest cumulative malaria incidence rate (38.1 cases/100,000 person-weeks). The 2009–2010 HTP had the lowest cumulative malaria incidence rate (7.53 cases/100,000 person-weeks) (Table 1).

**Table 1:** Characteristics of transmission periods and hotspots

Period: (cumulative inc^a^), dates, duration	Hotspot status	Number of villages	Cumulative incidence rate ^a^	SMC^b^ (%)	Weekly average rainfall (mm/week) (SD^c^)	Dominant vegetation type^d^ (% village)
2008 LTP (0.93) 01/01/2008 - 30/06/2008 - 26 weeks	Hotspot	19	9.82	0 (0.00%)	1.9 (0.99)	Mixed (63.16)
	Non-Hotspot	556	0.44	0 (0.00%)	1.6 (0.77)	Mixed (59.35)
2008–2009 HTP (14.88) 01/07/2008 – 12/01/2009 – 28 weeks	Hotspot	128	33.53	28 (21.87%)	26.41 (1.78)	Mixed (88.28)
	Non-Hotspot	447	5.46	47 (10.51%)	22.59 (2.46)	Mixed (51.23)
2009 LTP (0.88) 13/01/2009 – 03/08/2009 – 29 weeks	Hotspot	27	10.66	0 (0.00%)	10.54 (2.02)	Mixed (96.3)
	Non-Hotspot	548	0.5	0 (0.00%)	8.26 (1.57)	Mixed (83.58)
2009–2010 HTP (7.53) 04/08/2009 – 15/02/2010 – 28 weeks	Hotspot	62	27.04	23 (37.1%)	20.61 (1.79)	Mixed (93.55)
	Non-Hotspot	513	5.17	201 (39.18%)	18.21 (2.24)	Mixed (83.04)
2010 LTP (0.83) 16/02/2010 – 12/07/2010 – 21 weeks	Hotspot	22	12.61	0 (0.00%)	2.43 (1.46)	Mixed (68.18)
	Non-Hotspot	553	0.41	0 (0.00%)	3.39 (1.58)	Mixed (62.03)
2010–2011 HTP (29.9) 13/07/2010 – 24/01/2011 – 28 weeks	Hotspot	142	80.26	77 (54.22%)	23.4 (1.49)	Mixed (57.04)
	Non-Hotspot	433	19.61	294 (67.9%)	22.95 (2.98)	Mixed (63.97)
2011 LTP (2.73) 25/01/2011 – 22/08/2011 – 30 weeks	Hotspot	43	12.69	0 (0.00%)	8.39 (1.25)	Mixed (72.09)
	Non-Hotspot	532	1.57	0 (0.00%)	9.52 (0.83)	Grasslands (40.79)
2011–2012 HTP (14.62) 23/08/2011 – 09/01/2012 – 20 weeks	Hotspot	105	34.35	0 (0.00%)	18.52 (0.51)	Grasslands (35.24)
	Non-Hotspot	470	9.59	0 (0.00%)	18.93 (1.53)	Mixed (41.70)
2012 LTP (1.66) 10/01/2012 – 09/07/2012 – 26 weeks	Hotspot	10	19.16	0 (0.00%)	4.93 (0.69)	Mixed (60)
	Non-Hotspot	565	1.24	0 (0.00%)	3.61 (0.8)	Mixed (42.83)
2012 HTP (38.1) 10/07/2012 – 31/12/2012 – 24 weeks	Hotspot	147	119.24	0 (0.00%)	31.71 (2.36)	Grasslands (59.86)
	Non-Hotspot	428	25.74	0 (0.00%)	30.49 (1.93)	Mixed (51.17)

High Transmission Periods (HTP) and Low Transmission Periods (LTP) with cumulative incidence rate, start and end dates, and duration (in weeks); hotspot status of villages (hotspot or non-hotspot); number of hotspot and non-hotspot villages; cumulative incidence rate in hotspot and non-hotspot villages; number and percentage of hotspot and non-hotspot villages that received seasonal malaria chemoprevention (SMC); weekly average rainfall and standard deviation in hotspot and non-hotspot villages; dominant vegetation type (open shrublands, grasslands, croplands, mixed vegetation) in hotspot and non-hotspot villages for each period

^a^ Cumulative incidence rate (cases/100,000 person-weeks)

^b^ Number and percentage of villages that received SMC (seasonal malaria chemoprevention)

^c^ Standard deviation

^d^ Dominant vegetation type for each period

LTPs began in January or February and ended between June and August. The median LTP duration was 27.5 weeks. The 2011 LTP had the highest cumulative malaria incidence rate (2.73 cases/100,000 person-weeks), and the 2010 LTP had the lowest cumulative malaria incidence rate (0.83 cases/100,000 person-weeks) (Table 1).

### Hotspot characterization during HTPs

The cluster analysis helped to detect 356 villages (out of 575) that were malaria hotspots at least once during HTPs (Table 2).

**Table 2:** The different hotspot types and the associated number of villages

Hotspot type	Hotspot during all 5 LTPs	Hotspot mainly during HTPs	Hotspot mainly during LTPs	Hotspot equally during LTPs and HTPs	Never a hotspot	Hotspot only during HTPs	Hotspot only during LTPs
Number of villages (%)	3 (0.52%)	47 (8.17%)	5 (0.87%)	13 (2.26%)	205 (35.65%)	288 (50.1%)	14 (2.43%)

Number and percentage of villages that were a hotspot during all 5 LTPs, mainly during HTPs, mainly during LTPs, equally during LTPs and HTPs, never, only during HTPs, and only during LTPs

During HTPs, the median malaria incidence in hotspot and non-hotspot villages was 33.94 and 7.53 cases/100,000 person-weeks, respectively (Table 1).

**Fig. 2:**
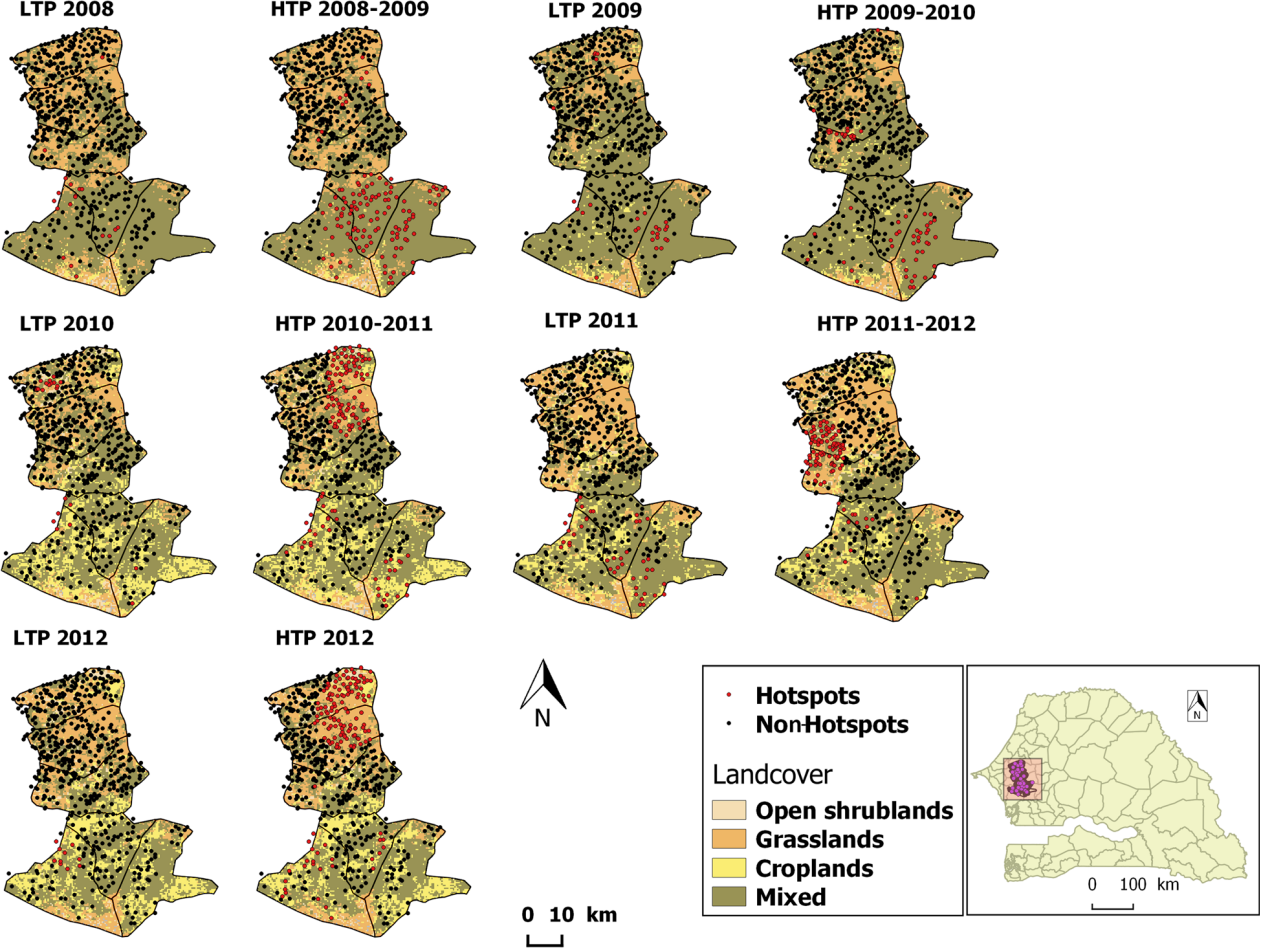
Spatio-temporal distribution of hotspot villages and vegetation type during transmission periods from 2008 to 2012. Hotspot villages (red dots) and non-hotspot villages (black dots); vegetation type (land cover: open shrublands in beige, grasslands in orange, croplands in yellow, and mixed vegetation in green) according to transmission periods (LTP, HTP) from 2008 to 2012 in Bambey and Fatick health districts, Senegal. Acknowledgements:the Senegal shapefile were downloaded in gadm website and Landcover rasters extracted from MODIS NASA website.

The 2012 HTP had the largest number of hotspot villages (147). These villages were mostly located in the northeast of the study area (Fig. 2) and were dominated by grasslands (representing 59.9% of villages). By contrast, the 428 non-hotspot villages were dominated by mixed vegetation (representing 51.2% of villages). This HTP showed the highest cumulative malaria incidence rate in both hotspot and non-hotspot villages (119.24 and 25.74 cases/100,000 person-weeks, respectively) (Table 1). It also showed the highest weekly average rainfall in both hotspot and non-hotspot villages (31.71 and 30.49 mm/week, respectively).

The 2009–2010 HTP was the least affected HTPs by malaria, with only 62 hotspot villages (37.1% of which received SMC intervention) compared to 513 non-hotspot villages (39.18% of which received SMC intervention). Hotspot villages were mainly located in the southeast of the study area (Fig. 2). The cumulative malaria incidence rate in hotspot and non-hotspot villages was 27.0 and 5.17 cases/100,000 person-weeks, respectively. The weekly average rainfall was low (but not the lowest) at 20.61 and 18.21 mm/week, respectively. Both hotspot and non-hotspot villages were dominated by mixed vegetation (representing 93.55 and 83.04% of villages, respectively).

### Hotspot characterization during LTPs

The cluster analysis helped to detect 82 villages (out of 575) that were malaria hotspots at least once during LTPs (Table 2).

During LTPs, the median malaria incidence in hotspot and non-hotspot villages was 12.65 and 0.87 cases/100,000 person-weeks, respectively (Table 1).

The 2011 LTP had the longest duration (30 weeks) and showed the highest number of hotspot villages (43). These villages were located mainly in the south of the study area (Fig. 2). This LTP showed a high cumulative malaria incidence rate in hotspot villages and the highest cumulative malaria incidence rate in non-hotspot villages (12.69 and 1.57 cases/100,000 person-weeks, respectively). The weekly average rainfall was fairly high at around 9 mm/week in both hotspot and non-hotspot villages. Hotspot villages were dominated by mixed vegetation (representing 72.09% of villages), whereas non-hotspot villages were dominated by grasslands (representing 40.79% of villages).

The 2010 LTP had the shortest duration (21 weeks) and 22 hotspot villages located in the northwest and west-central parts of the study area (Fig. 2). The cumulative malaria incidence rate in hotspot villages was 12.61 cases/100,000 person-weeks, compared to a very low cumulative malaria incidence rate of 0.41 cases/100,000 person-weeks in the 553 non-hotspot villages. The weekly average rainfall was low at around 3 mm/week in both hotspot and non-hotspot villages.

The descriptions of the other transmission periods are available in additional file 2.

### Factors associated with the spatio-temporal variation of malaria hotspots

According to the multivariate analysis (GAMM, 38% deviance explained), villages receiving SMC intervention were protected from the risk of being a hotspot (OR = 0.48, 95%CI: (0.33, 0.68)). The random effect of health posts was significant (τ = 0.53, 95%CI: (0.31, 0.88)).

**Fig. 3:**
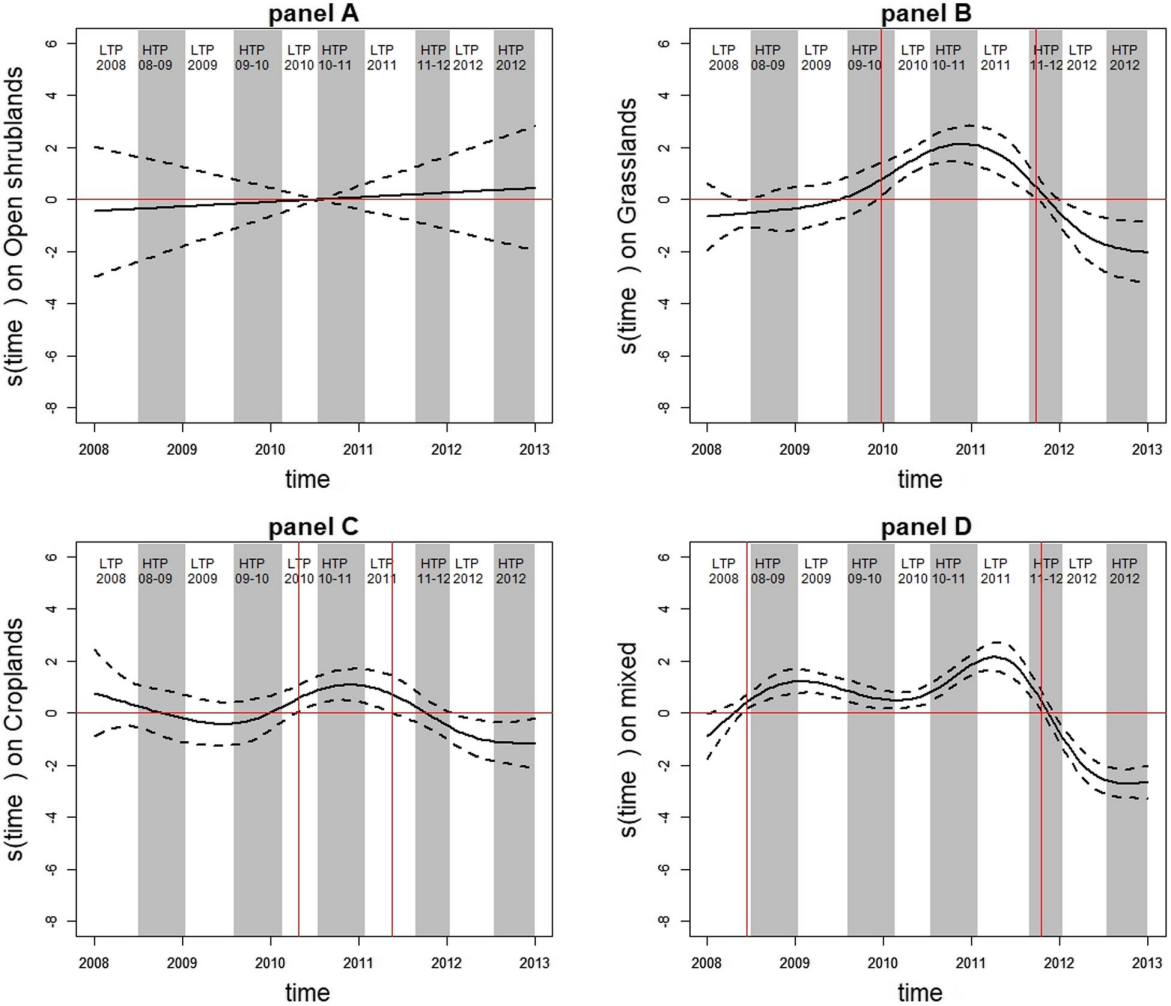
Temporal evolution of the risk of being hotspot with 95%CI according to vegetation type. Temporal evolution of the risk of being a hotspot (continuous black curve) with 95% confidence interval (discontinuous black curves) according to each vegetation type: open shrublands (panel **a**), grasslands (panel **b**), croplands (panel **c**), and mixed vegetation (panel **d**). HTPs and LTPs are indicated in grey and white, respectively. The vertical red lines indicate the dates of interest, at which villages were at risk of being hotspot. The horizontal red lines indicate the zero reference line

**Fig. 4:**
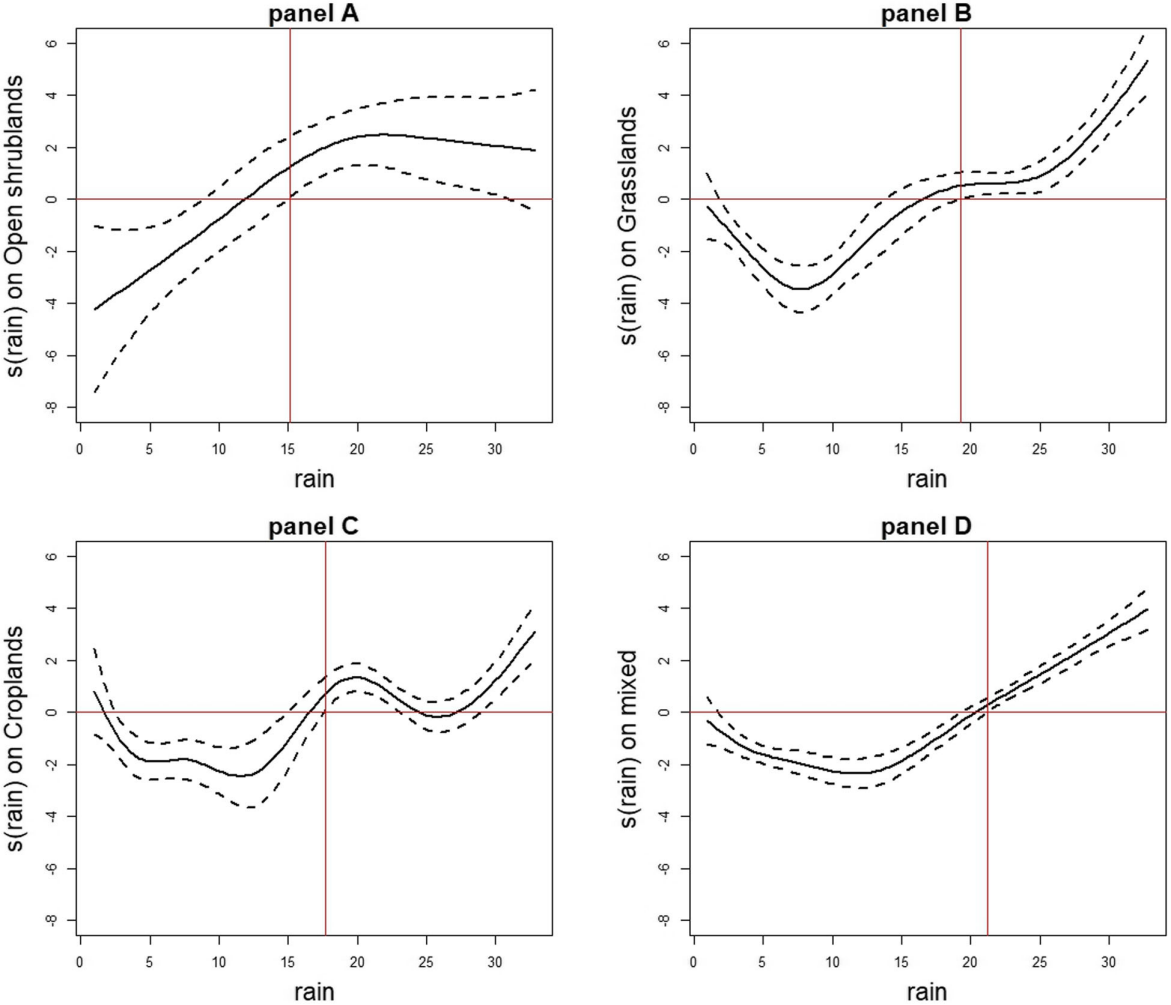
Evolution of the risk of being hotspot with 95%CI according to rainfall and vegetation type. Evolution of the risk of being a hotspot (continuous black curve) with 95% confidence interval (discontinuous black curves) according to weekly rainfall and vegetation type: open shrublands (panel **a**), grasslands (panel **b**), croplands (panel **c**), and mixed vegetation (panel **d**). Vertical red lines show the amount of rainfall starting from which rainfall became a risk factor

For villages dominated by open shrublands, the risk of being a hotspot did not vary over time (Fig. 3, panel A). A non-linear association was found between rainfall and the risk of being a hotspot (*p* = 0.0002; Fig. 4, panel A). When rainfall was not very abundant, these villages were relatively protected from the risk of being a hotspot. However, this risk became significant from 15 mm/week rainfall (Smoothed value = 1.26, 95%CI: (0.09, 2.43)) and continued to increase before stabilizing at a maximum rainfall of around 22 mm/week (Smoothed value = 2.47, 95%CI: (1.24, 3.7)).

For villages dominated by grasslands, the risk of being a hotspot varied significantly over time (*p* < 0.0001; Fig. 3, panel B). This risk became significant and increased from late December 2009 (Smoothed value = 0.76, 95%CI: (0.12, 1.40)), peaked in early November 2010 (Smoothed value = 2.13, 95%CI: (1.45, 2.82)), and then decreased until late September 2011 (Smoothed value = 0.46, 95%CI: (0.03, 0.9)). These villages were protected from the risk of being a hotspot from early January 2012 (Smoothed value = − 0.65, 95%CI: (− 1.2, − 0.1)) to late December 2012 (Smoothed value = − 2.04, 95%CI: (− 3.22, − 0.84)). Moreover, a non-linear association was found between rainfall and the risk of being a hotspot (*p* < 0.0001; Fig. 4, panel B). When rainfall was not very abundant, these villages were relatively protected from the risk of being a hotspot. However, this risk became significant from 19 mm/week rainfall (Smoothed value = 0.52, 95%CI: (0.01, 1.04)) and increased with rainfall.

For villages dominated by croplands, the risk of being a hotspot varied little over time (*p* = 0.0013; Fig. 3, panel C). This risk became significant from late April 2010 (Smoothed value = 0.58, 95%CI: (0.07, 1.08)), peaked in mid-November 2010 (Smoothed value = 1.08, 95%CI: (0.48, 1.68)), and then decreased until mid-May 2011 (Smoothed value = 0.7, 95%CI: (0.01, 1.42)). These villages were relatively protected from the risk of being a hotspot from late January 2012 to late December 2012 (Smoothed value = − 0.63, 95%CI: (− 1.2, 0.05) to Smoothed value = − 1.17, 95%CI: (− 2.12, − 0.25)). Moreover, a non-linear association was found between rainfall and the risk of being a hotspot (*p* < 0.0001; Fig. 4, panel C). When rainfall was not very abundant, these villages were relatively protected from the risk of being a hotspot. However, this risk became significant from 18 mm/week rainfall (Smoothed value = 0.72, 95%CI: (0.09, 1.36)) and continued to increase roughly with rainfall.

For villages dominated by mixed vegetation, the risk of being a hotspot varied over time (*p* < 0.0001; Fig. 3, panel D). This risk increased from mid-June 2008 (Smoothed value =0.42, 95%CI: (0.14, 0.7)), peaked in early April 2011 (Smoothed value =2.16, 95%CI: (1.61, 2.71)), and then decreased until mid-October 2011 (Smoothed value =0.48, 95%CI: (0.09, 0.87)). These villages were protected from the risk of being a hotspot from late November 2011 (Smoothed value = − 0.4, 95%CI: (− 0.76, − 0.03)) to late December 2012 (Smoothed value = − 2.65, 95%CI: (− 3.22, − 1.97)). Moreover, a non-linear association was found between rainfall and the risk of being a hotspot (*p* < 0.0001; Fig. 4, panel D). Once again, when rainfall was not very abundant, the villages were relatively protected from the risk of being a hotspot. This risk became significant from 21 mm/week rainfall (Smoothed value = 0.29, 95%CI: (0.02, 0.56)) and continued to increase linearly with rainfall.

**Fig. 5:**
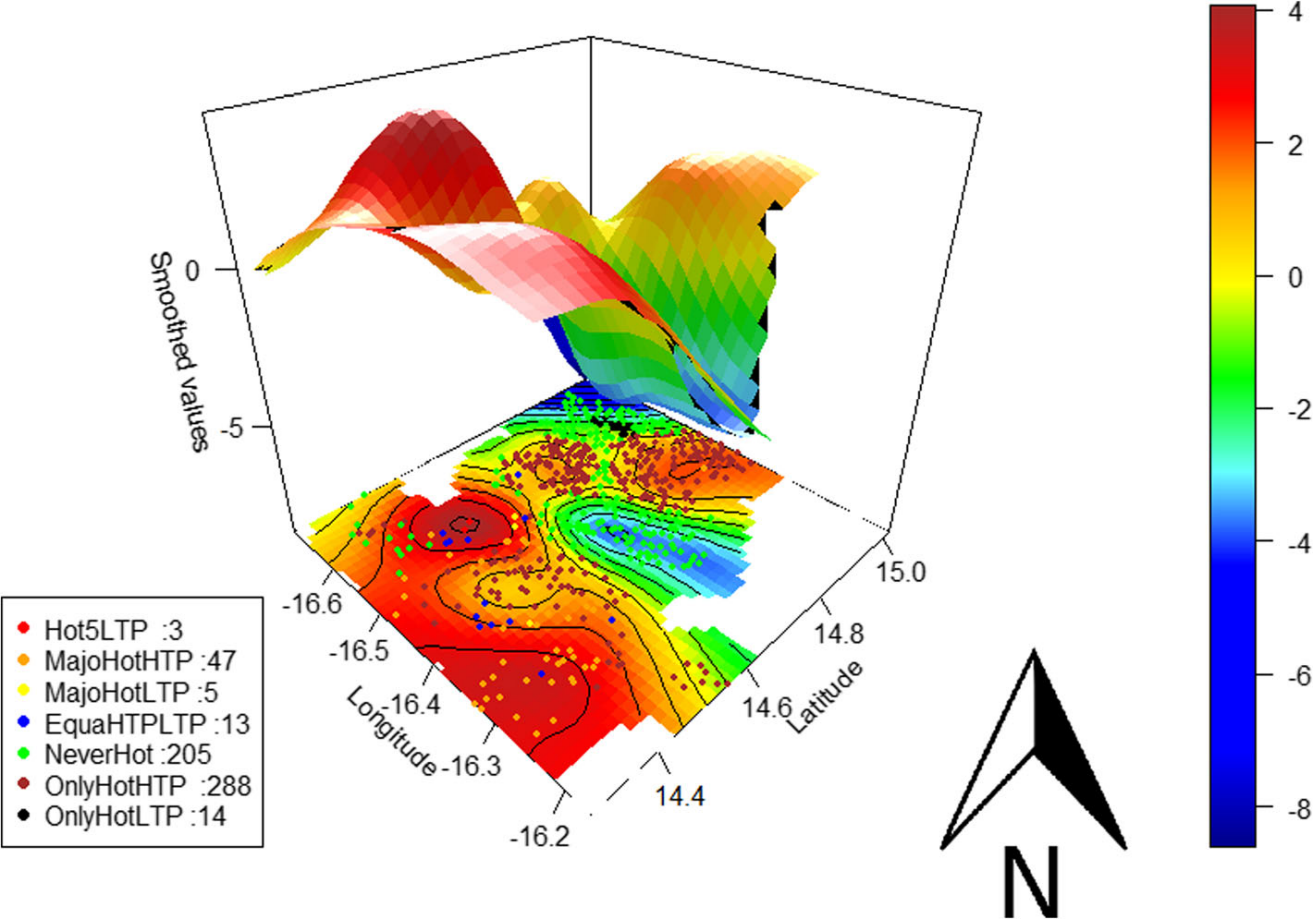
Spatial distribution of the different hotspot types and the associated spatial risk of being hotspot. The black curves are the contours of bivariate smoothed values; the colour bar is the ascending level of risk indicated by the spline smoothing function values (smoothed values) from blue to red; red dots represent the villages that were a hotspot during all 5 LTPs (Hot5LTP), orange dots those that were a hotspot mainly during HTPs (MajoHotHTP), yellow dots those that were a hotspot mainly during LTPs (MajoHotLTP), blue dots those that were a hotspot equally during HTPs and LTPs (EquaHTPLTP), green dots those that were never a hotspot (NeverHot), brown dots those that were a hotspot only during HTPs (OnlyHotHTP), and black dots those that were a hotspot only during LTPs (OnlyHotLTP)

According to the spatial interpolation obtained with the multivariate GAMM for the entire study (Fig. 5), 2 zones (red colour) located in the southwest and southeast of the study area had the highest risk of being a hotspot (Smoothed value min = 0.64, 95%CI: (0.02, 1.27); Smoothed value max = 4.1, 95%CI: (3.29, 4.92)). Moreover, villages located in 2 geographically restricted areas—one in the extreme northwest and the other in the east-central part of the study area (blue colour)—were relatively protected from this risk (Smoothed value min = − 8.99, 95%CI: (− 12.96, − 5.02); Smoothed value max = − 0.8, 95%CI: (− 1.57, − 0.03)).

## Discussion

In our study, the risk of a village being a malaria hotspot varied over time and space, depending on meteorological, environmental, and preventive factors. Two malaria transmission periods types were identified, with HTPs extending from July–August to January–February of the next year, well after the end of the rainy season. Similar transmission dynamics have been reported in Mali (Bamako and Bandiagara) [15, 35] and Burkina Faso (Ouagadougou) [36].

Malaria persisted in the study area during LTPs (with a low to very low incidence), as only 5 weeks showed no recorded cases (3 non-consecutive weeks in 2009, 1 week in 2010, and 2012). This confirms that malaria was endemic in the study area.

Our findings indicate that the temporal dynamics of malaria incidence should be taken into consideration in studies of malaria in the Sahel. Moreover, they highlight the importance of collecting data beyond the end of the rainy season, as opposed to aggregating them by calendar year. The latter approach fails to accurately represent HTPs, and may therefore hinder the effectiveness of control strategies. In our study, the last HTP was slightly incomplete due to the fact that data were collected from January 1st, 2008 to December 31st, 2012.

While hotspots have enjoyed renewed interest since the 2000s [37], there is no consensus on their definition or on the method that should be adopted for their detection [8, 21, 38, 39]. Our study used the statistical definition given by the Kulldorff cluster detection method [31]. Because the performances of this method are known to be sensitive to edge effects and non-circular clusters [40, 41], an elliptical window was used to minimize this impact and the Oliveira measure was used to assess the cluster edge [42]. However, 14 villages located in the southwest of the study area were never identified as hotspot villages in our cluster analysis, even though spatial interpolation obtained with the multivariate GAMM found that the risk of being a hotspot was high in this zone (Fig. 5). Knowing that SaTScan performance improves with incidence level, size of at-risk population, and relative risk [43, 44], we divided the malaria incidence rate time series into HTPs and LTPs with the change point analysis method, before assessing spatial clusters to facilitate the detection of hotspot villages during low transmission periods. In addition, in a highly seasonal transmission setting, the dynamics of malaria incidence can vary greatly between the seasonal peak (during which incidence is expected to increase) and the low-transmission season (during which incidence can persist at low intensity). As expected, the spatial location of cases was more spread out in the high incidence season than in the low incidence season due to favourable conditions for transmission. While the analysis of high transmission periods allows for identifying zones with the highest burden of cases, a separate analysis of low transmission periods may help to detect foci of persistent transmission that can play a significant role in the annual resurgence of epidemics. These elements could help the Senegal malaria program to refine its targeted control strategies. Moreover, our study area covers part of the health districts defined as low transmission (incidence < 5 cases/ 1000 person-years) by the Senegal malaria program. This program has already focused on hotspots: focal test and treat, focal screen and test, focal drug administration, epidemic response indoor residual spraying, primaquine single low-dose [45]. It should be noted that our approach is similar to that used in other studies conducted in Mali and Burkina Faso [35, 36, 46].

Hotspot variation is not obvious. In our study, the location of hotspots was unstable across transmission periods (LTPs and HTPs). Seasonal and annual instability of malaria hotspots (household and village scales) was also reported in Kenya and in Sudan (Khartoum) [3, 7, 8, 21, 47]. By contrast, malaria hotspots were found to be relatively stable in Burkina Faso (Ouagadougou and Nanoro) and Mali [15, 36, 46], while *P. falciparum* carrier hotspots were shown to be stable in Kenya [8]. The data on parasite carriage were not available in our study. As a result, we were unable to explore the relationship between hotspot, the force of infection, and clinical incidence, as was done in other studies [8].

Our study found non-linear associations between meteorological, environmental, and preventive factors. It also found that the risk of being a hotspot varied over time and space and according to health post (significant random effect).

We therefore sought to explore how the variation of factors (e.g., meteorological and environmental factors) impacted the variation in the risk of a village being a hotspot over time and space. Our results showed that rainfall was positively associated with the risk of being a hotspot, and this non-linear association depended on vegetation type. While the relationship between rainfall and malaria occurrence has been widely discussed in the literature [15, 35, 36, 48, 49], our study indicates that the impact of rainfall on malaria depends on both the amount of rainfall and the type of vegetation, and that this interaction in turn modifies hotspot distribution. Thus, for villages dominated by open shrublands, the risk of being a hotspot increases from the first rains and then reaches a plateau from 22 mm/week, likely because heavy rains destroy breeding sites [50, 51]. By contrast, for villages dominated by grasslands, croplands, or mixed vegetation, the risk of being a hotspot increases only when rainfall is above 10 or 15 mm/week. The low smoothed values corresponding to the beginning of the curves (Fig. 4, panel C) may be explained by soil quality or ploughing practices that increase water infiltration [52] and reduce breeding sites. In view of the spatio-temporal instability of hotspots, we attempted to identify the similar characteristics of hotspots based mainly on variations in environmental and meteorological factors, as these contribute to the spatial heterogeneity of malaria. These factors should be monitored [53] taking into consideration the environmental and meteorological characteristics common to all hotspots, as this would lead targeted public health interventions at the local level.

Moreover, in our study, the risk of being a hotspot varied according to the geographical location of villages (Fig. 5), which confirms results from studies in India [54], Kenya [21], and Ghana [55]. Thus, almost all of the villages that were never a hotspot during the 10 transmission periods were located in the 2 zones with the lowest risk, i.e., in the northwest and east-central part of the study area (Fig. 5). While the risk of being a hotspot was highly variable in the south, 3 villages located in the highest risk zone were hotspot during all LTPs. These hotspots that persist during LTPs may be the source of the seasonal increase in malaria transmission [3, 5, 56]. Furthermore, we were unable to assess the source of transmission or the reservoir of infections because we lacked data on mobility and asymptomatic parasite carriers [57], but they are an important and increasingly reported source of malaria in low transmission areas [39, 58]. These factor may contribute to the instability of hotspots over space and time.

Lastly, our study found that villages receiving SMC intervention were protected from the risk of being a hotspot, corroborating studies that highlighted the effectiveness of SMC interventions in Senegal [16, 59]. Yet, despite the implementation of malaria control strategies combining SMC, mass drug administration, long-lasting insecticide-treated nets, and indoor residual spraying [6, 59–62], malaria incidence remains high in the country [2]. Malaria control strategies are generally implemented at the beginning or in the middle of the rainy season [16, 59–62], which effectively corresponds to HTPs. However, as our findings suggest, the increase in malaria incidence in hotspot villages and persisting hotspots observed during LTPs can also affect malaria transmission during HTPs. In view of this, we recommend that spatially targeted strategies identifying transmission bottlenecks be further addressed during LTPs, as this may help shrink the parasite reservoir and may thereby prevent malaria transmission during subsequent HTPs.

### Strengths

- We worked on quality data from a randomized trial using a very fine and precise and spatio-temporal scale.
- Using a set of spatial and temporal analysis methods, our study proposed a methodology that explained the variation of malaria hotspots over time and space. This allowed us to estimate the risk of a village being a hotspot anytime and anywhere in the study area.
- This study allowed also to assess the impact of the interaction between rainfall and vegetation on the risk of a village being a hotspot. Therefore, we obtained an estimate of the risk of a village being a hotspot dependent on amount of rainfall and vegetation type. This estimate could be made for any other factor.

### Limitations

- Data on rainfall and vegetation types were not observed but were obtained through remote sensing. Some villages received therefore the same amount of rainfall as their neighbours due to pixel resolution.
- Socio-economic and behaviour data, which could have helped to further explain the variation of hotspots, were not available.
- The source of transmission or infectious reservoir could not be assessed because data on mobility and asymptomatic parasite carriers were unavailable. This lack of data also prevented the assessment of the relationship between hotspot, force of infection, and clinical incidence, which has been explored in other studies.

## Conclusions

This study highlights the important variability (even at a very local scale) of malaria transmission in central Senegal over space and time, as well as the impact of meteorological, environmental, and protective factors on malaria risk. These factors should be monitored taking into consideration the environmental and meteorological characteristics common to all hotspots, as this would lead targeted public health interventions at the local level. Moreover, spatial hotspots and foci of malaria persisting during LTPs need to be further addressed.

## Supplementary information

**Additional file 1.** Vegetation type for each village determination: A 0.55 km radius buffer zone is defined around a village (light blue point) in 2012. Each colour represents a vegetation type: open shrublands (beige, 67.2%), grasslands (orange, 26.2%), croplands (yellow, 3.1%), and mixed vegetation (green, 3.5%). Thus, the dominant vegetation type for this village in 2012 is open shrublands.

**Additional file 2.** The descriptions of the other transmission periods: 2010–2011 HTP, 2011–2012 HTP, 2008–2009 HTP, 2012 LTP, 2009 LTP and 2008 LTP.

## Data Availability

The datasets analysed in this study are available from the corresponding author on reasonable request. Moreover, a request of raw data is possible with this reference: Milligan, P (2016). Effectiveness of Seasonal Malaria Chemoprevention in children under 10 years of age in Senegal: a stepped-wedge cluster-randomized trial. [Data Collection]. London School of Hygiene & Tropical Medicine, London, United Kingdom. 10.17037/DATA.117

## Abbreviations

GAMM: Generalized Additive Mixed Model
GPS: Global Positioning System
HTP: High Transmission Period
LTP: Low Transmission Period
MBIC: Modified Bayes Information Criterion
MODIS: Moderate Resolution Imaging Spectroradiometer
NASA: National Aeronautics and Space Administration
OR: Odd-Ratio
PELT: Pruned Exact Linear Time
SMC: Seasonal Malaria Chemoprevention
TRMM: Tropical Rainfall Measuring Mission
WHO: World Health Organization
