# Supplementary figures and images for: Spatio-temporal variation of malaria hotspots in Central Senegal, 2008–2012
Source: BMC Infect Dis. 2020 Jun 17;20:424. doi: 10.1186/s12879-020-05145-w (PMC7301493; doi:10.1186/s12879-020-05145-w)

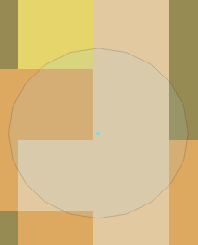

Supplement: Supplementary file 1 — Additional file 1. Vegetation type for each village determination: A 0.55 km radius buffer zone is defined around a village (light blue point) in 2012. Each colour represents a vegetation type: open shrublands (beige, 67.2%), grasslands (orange, 26.2%), croplands (yellow, 3.1%), and mixed vegetation (green, 3.5%). Thus, the dominant vegetation type for this village in 2012 is open shrublands. [file 12879_2020_5145_MOESM1_ESM.tiff]
