# Supplementary material for: Spatio-temporal variation of malaria hotspots in Central Senegal, 2008–2012
Source: BMC Infect Dis. 2020 Jun 17;20:424. doi: 10.1186/s12879-020-05145-w (PMC7301493; doi:10.1186/s12879-020-05145-w)
Supplement: Supplementary file 2 — Additional file 2. The descriptions of the other transmission periods: 2010–2011 HTP, 2011–2012 HTP, 2008–2009 HTP, 2012 LTP, 2009 LTP and 2008 LTP. [file 12879_2020_5145_MOESM2_ESM.docx]

The 2010-2011 HTP showed 142 hotspot villages (54.22% of which received SMC intervention) and 433 non-hotspot villages (67.9% of which received SMC intervention). Hotspot villages were mainly located in the northeast of the study area (Figure 2). The cumulative malaria incidence in hotspot and non-hotspot villages was high at 80.26 and 19.61 cases/100,000 person-weeks, respectively. Similarly, the weekly average rainfall was high in both hotspot and non-hotspot villages (23.4 and 22.94 mm/week, respectively). Both hotspot and non-hotspot villages were dominated by mixed vegetation (representing 57.04% and 63.97% of villages, respectively).

The 2011-2012 HTP had the shortest duration (20 weeks) and the lowest weekly average rainfall (around 19 mm/week in both hotspot and non-hotspot villages). The cumulative malaria incidence rate remained fairly low in both hotspot and non-hotspot villages (34.4 and 9.6 cases/100,000 person-weeks, respectively). The number of hotspot and non-hotspot villages were 105 and 470 respectively. No SMC was implemented during this HTP.

The 2008-2009 HTP was characterized by a very large hotspot that included 128 villages covering a large zone located in the south of the study area (Figure 2). The cumulative malaria incidence rate in hotspot and non-hotspot villages was 33.53 and 5.46 cases/100,000 person-weeks, respectively. SMC was implemented in 21.87% of hotspot villages and in 10.51% of non-hotspot villages. Both hotspot and non-hotspot villages were dominated by mixed vegetation (representing 88.28% and 51.23% of villages, respectively). The weekly average rainfall was fairly high both in hotspot and non-hotspot villages (26.41 and 22.59 mm/week, respectively).

The 2012 LTP showed the lowest number of hotspot villages (10). These villages were located in the southwest of the study area (Figure 2). The cumulative malaria incidence in hotspot villages was the highest at 19.16 cases/100,000 person-weeks; by contrast, the cumulative malaria incidence in the 565 non-hotspot villages was 1.24 cases/100,000 person-weeks. Both hotspot and non-hotspot villages were dominated by mixed vegetation (representing 60% and 42.83% of villages, respectively). The weekly average rainfall was fairly high at around 4 mm/week in both hotspot and non-hotspot villages.

The 2009 LTP was characterized by relatively high weekly average rainfall in both hotspot and non-hotspot villages (10.5 mm/week and 8.26 mm/week, respectively). Despite this abundant rainfall, the cumulative malaria incidence rate was 10.66 cases/100,000 person-weeks in the 27 hotspot villages and 0.5 cases/100,000 person-weeks in the 548 non-hotspot villages. The hotspot villages were located in the south of the study area.

The 2008 LTP had 19 hotspot villages located in the south of the study area (Figure 2). It showed the lowest cumulative malaria incidence rate (9.82 cases/100,000 person-weeks) and very low rainfall (1.9 mm/week) in hotspot villages, compared to 0.44 cases/100,000 person-weeks and 1.6 mm/week in the 556 non-hotspot villages.
